# Notalgia Paresthetica Responding Positively to Chiropractic Spinal Manipulation: A Case Report

**DOI:** 10.7759/cureus.53382

**Published:** 2024-02-01

**Authors:** Robert J Trager, Curtis P Riffle, Cliff Tao

**Affiliations:** 1 Chiropractic, Connor Whole Health, University Hospitals Cleveland Medical Center, Cleveland, USA; 2 Family Medicine and Community Health, Case Western Reserve University School of Medicine, Cleveland, USA; 3 Biostatistics and Bioinformatics Clinical Research Training Program, Duke University School of Medicine, Durham, USA; 4 Radiology, Private Practice of Chiropractic Radiology, Irvine, USA

**Keywords:** case report, pruritus, notalgia paresthetica, spinal manipulation, chiropractic

## Abstract

Notalgia paresthetica (NP) is a chronic cutaneous neuropathy characterized by localized pruritus and pain, numbness, and/or paresthesia, often linked to degenerative cervicothoracic changes. Treatment options for NP are limited. This case report details a 54-year-old woman with a six-year history of right-sided periscapular pruritus and cervicothoracic discomfort who presented to a chiropractor upon referral with a prior diagnosis of NP. Prior topical treatments yielded minimal relief. Radiographs revealed degenerative spinal changes at C5/6 and C6/7 which correlated with her periscapular symptom distribution. The patient responded positively to chiropractic spinal manipulative therapy (SMT), focusing on the cervicothoracic region, coupled with myofascial release. Symptoms significantly improved after a single SMT session and resolved after a second session, with no pruritus returning over one-month follow-up. While this case highlights the potential benefits of SMT for NP, further research is needed to explore the effectiveness of this treatment.

## Introduction

Notalgia paresthetica (NP) is a chronic cutaneous neuropathy characterized by localized pruritus and associated pain, numbness, and/or paresthesia, affecting the medial or inferior scapular border [1]. It is classified as a type of neuropathic itch, which is an itch caused by a lesion of or disease of the somatosensory nervous system [2]. NP is often associated with degenerative changes in the cervical and/or thoracic spinal joints, yet definitive mechanisms leading to pruritus have not been elucidated [1,3]. In addition, treatment for NP remains underexplored [1].

Diagnosis of NP is clinical and may be supported by radiological evidence of degenerative cervical or thoracic changes [1,2]. A diffuse pattern of pruritus is inconsistent with NP and should prompt evaluation for concurrent metabolic, gastrointestinal, cancerous, or infectious disorders [2]. NP is more common in women at least age 40, with a female-to-male ratio of 2-3:1, and is usually unilateral [1]. Chronic scratching may lead to a hyperpigmented patch in the affected area in up to two-thirds of patients yet is not a required feature for NP diagnosis [3].

Without a definitive treatment for NP, clinicians often use a multidisciplinary approach including topical agents (e.g., botulinum toxin A, capsaicin, lidocaine, and steroids), oral gabapentinoids, and physical therapy exercises [1,3]. A recent clinical trial showed that difelikefalin, a selective kappa opioid receptor agonist, was moderately efficacious yet presented the risk of adverse events such as dizziness and nausea [4]. In addition, the use of gabapentin carries the risk of rebound pruritus [2].

Spinal manipulative therapy (SMT) is a safe and effective treatment for back pain [5,6]. However, research regarding the effectiveness of SMT for NP is very limited. A literature search using PubMed and Google Scholar on January 4, 2024, with derivations of the terms “chiropractic,” “spinal manipulation,” and “notalgia paresthetica” and review article [7] revealed only two cases of NP that improved with SMT [8,9].

Given the limited available treatments for NP, we describe a case of an adult with chronic NP who responded positively to SMT.

## Case presentation

**Figure 1:**
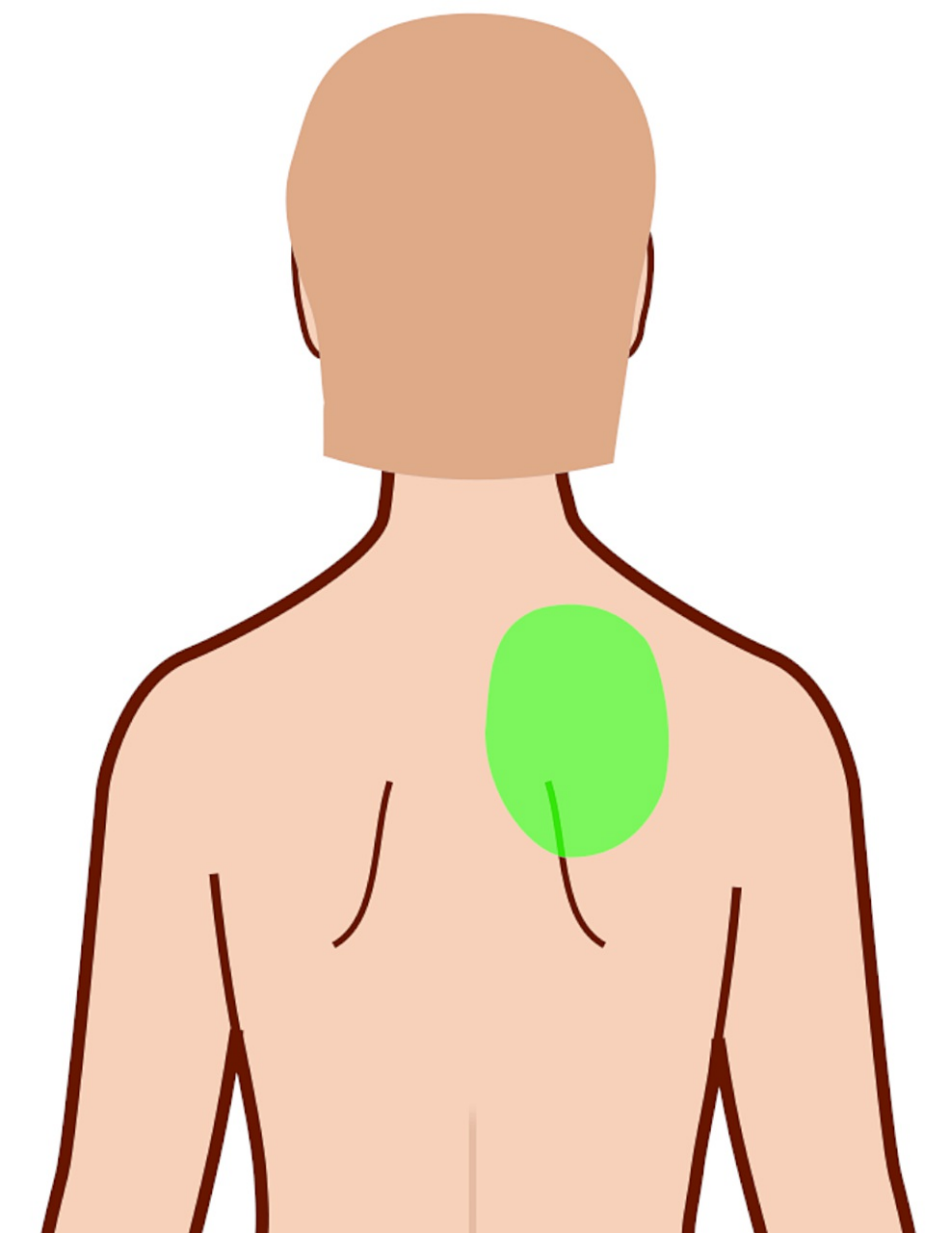
Distribution of the patient’s pruritus (green oval). Symptoms extended from the right interscapular region to the right superior scapular angle. Shading added to Wikimedia image from Jmarchn using the Creative Commons Attribution-Share Alike 3.0 Unported license.

A 54-year-old woman presented to a chiropractor with a six-year history of constant right periscapular pruritus which persisted at an average intensity of 4 out of 10 on the numeric rating scale (Figure 1). She also endorsed tightness affecting the neck and cervical thoracic regions, predominantly on the right side. Prior treatments included topical creams and lotions with little results. There were no specific factors that she noticed which increased or reduced symptoms. The patient exercised at least five times per week including strength training, aerobic exercise, and yoga. She rated her overall health as ‘excellent’ yet did endorse fatigue. She denied any history of Herpes zoster, diabetes, or chronic kidney disease. The patient had recently been evaluated by a dermatologist who diagnosed her with NP and referred her to a chiropractor. This provider suspected that the patient’s symptoms were caused by a spinal nerve issue.

Her medical history was significant for uveitis, periorbital pain, floaters, and partial hearing loss which had been diagnosed as Vogt-Koyanagi-Harada (VKH) syndrome based on extensive ophthalmologic and audiologic testing. This condition is a central nervous system disorder that affects vision and hearing yet is not typically associated with neuropathic itch [10]. The patient’s orbital symptoms arose six months prior to the current visit yet had improved under the management of an ophthalmologist and rheumatologist, as she was initially prescribed an oral steroid and then was transitioned to mycophenolic acid 500 mg twice daily.

Over the year leading up to the chiropractic visit, the patient underwent extensive laboratory testing in relation to her pruritis and eye symptoms which were all within normal limits, including acid fast bacilli blood culture, antinuclear antibody, Bartonella henselae antibody, C-reactive protein, complete blood count, comprehensive metabolic panel, cytological examination of the cerebrospinal fluid, erythrocyte sedimentation rate, hepatitis B and C antibodies, human immunodeficiency virus antibody, Lyme disease polymerase chain reaction, rapid plasma reagin, rheumatoid factor, T-spot tuberculosis, and thyroid stimulating hormone. This testing helped exclude other systemic disorders and etiologies of pruritus.

Examination revealed a rounded shoulder position and increased thoracic kyphosis. There was no evident hyperpigmentation or excoriation. Active and passive lateral neck flexion was reduced bilaterally, and active thoracic spine extension was reduced. Upper and lower extremity motor strength, muscle stretch reflexes, and sensation were normal. On palpation, there was hypertonicity and tenderness affecting the cervical paraspinal muscles, right scalenes, sternocleidomastoid, upper trapezius, levator scapulae, rhomboids, posterior shoulder girdle, thoracic paraspinals, and pectoralis minor, all predominantly on the right side. Limited joint mobility was evident on motion palpation of the C4/5, C5/6, C7/T1 and T3/4, T4/5, T5/6 articulations. Foraminal compression and cervical distraction tests did not affect the patient’s symptoms.

**Figure 2:**
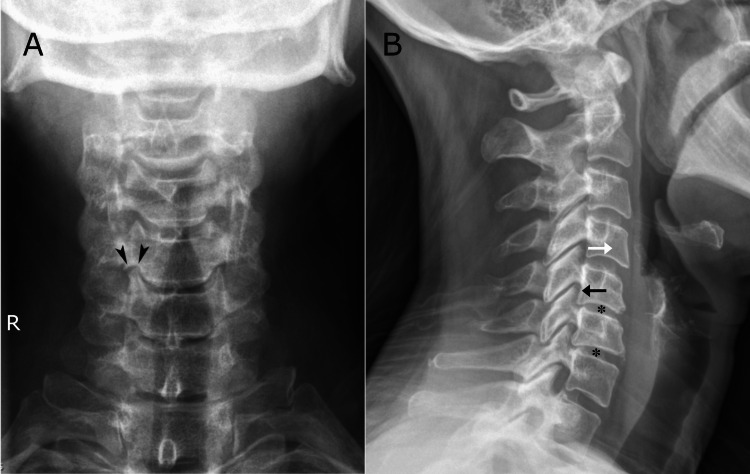
Anteroposterior (A) and lateral (B) cervical spine radiographs. Prominent degenerative uncovertebral arthrosis at C5/6 on the right (R), forming a “roof” (arrowheads) over the uncinate process, not seen at other levels. On the lateral view, moderate disc degeneration is evident at C5/6 and C6/7 (asterisks; *) while there is a subtle anterolisthesis of C4 (white arrow) and subtle retrolisthesis of C5 (black arrow).

**Figure 3:**
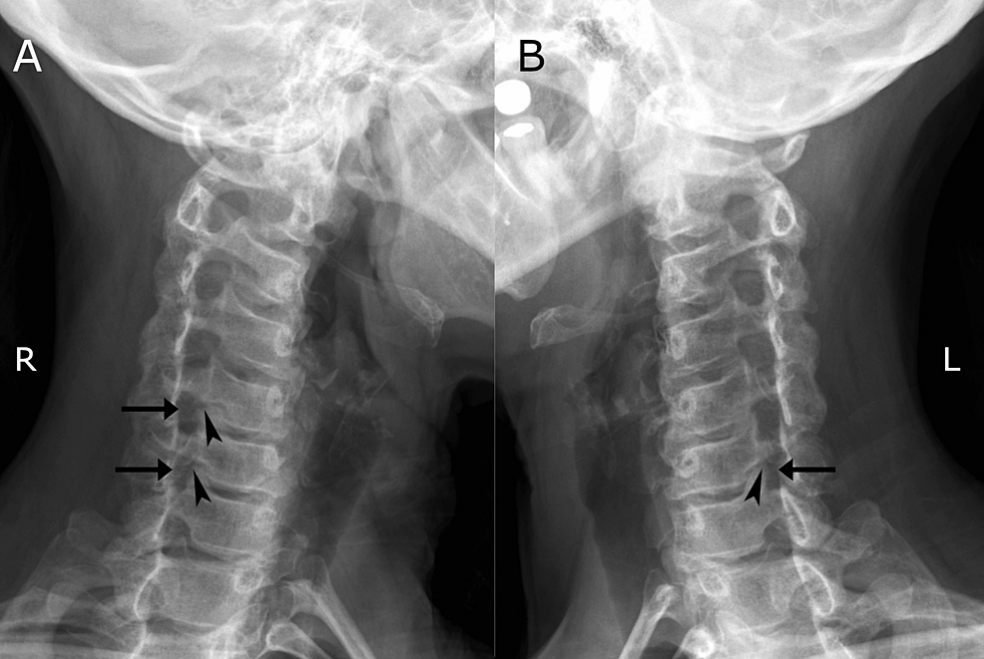
Anterior oblique cervical radiographs comparing right (A) and left (B) intervertebral foramina. Uncinate process hypertrophy is indicated by arrowheads while foraminal stenosis is indicated by arrows. Foraminal stenosis is evident at C5/6 and C6/7 on the right and at C6/7 on the left in relation to uncinate hypertrophy. The side of the patient closest to the film is indicated by the marker (right [R] or left [L]) which corresponds with the side of intervertebral foramina visualized.

Due to the patient’s autoimmune disorder (VKH), chronic symptoms, and chronic and persistent NP symptoms, the chiropractor deferred treatment on the first visit and ordered cervical spine radiographs, which revealed a trace anterolisthesis of C4 and trace retrolisthesis of C5, moderate disc degeneration at C5/6 and C6/7 (Figure 2), and foraminal stenosis at C5/6 and C6/7 on the right and C6/7 on the left (Figure 3).

The chiropractor concurred with the patient’s previous diagnosis of NP for two reasons: (1) prior testing had excluded other common causes of pruritus (e.g., metabolic, infectious) and (2) radiographs revealed degenerative spondylosis predominantly affecting C5/6 and C6/7 with foraminal stenosis affecting these two levels on the right side, corresponding with the C6 and C7 nerve roots. Awareness of the C6 and C7 nerve root periscapular distribution reinforced the correlation between imaging findings and her region of pruritus [11,12]. Dorsal scapular neuropathy was considered unlikely given the absence of numbness and scapular weakness or winging.

The patient returned a week later and consented to a treatment plan including SMT and myofascial release. The chiropractor performed prone high-velocity, low-amplitude manipulation to the cervical thoracic junction and hypomobile thoracic segments (Figure 3) and performed myofascial release to the right levator scapulae and pectoralis minor muscles. Two days later, the patient sent a message via a secure chart portal that her symptoms of neck tightness and pruritus were almost completely resolved. The patient returned a week later reporting that a reduction in symptoms had lasted. The same treatment was administered, which was tolerated well. Due to the significant improvement in symptoms, the patient’s care was hereafter provided on an as-needed basis.

**Figure 4:**
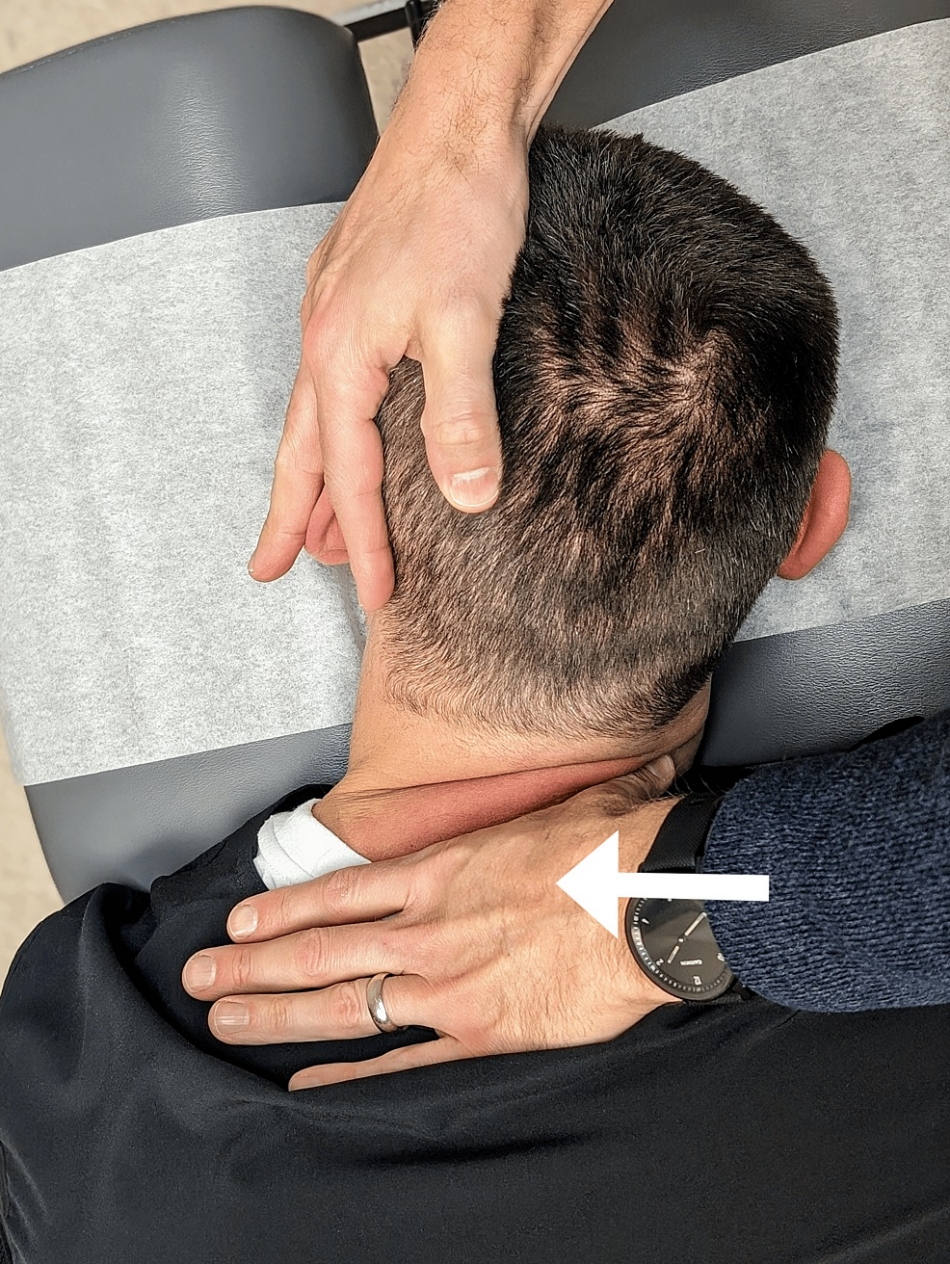
Prone cervicothoracic manipulation. The chiropractor places their thumb-web space at the lateral aspect of the spinous process and provides a high-velocity, low-amplitude thrust medially and slightly anteriorly (arrow), while the non-thrusting hand acts to stabilize the head with the neck laterally flexed towards the side of contact. In the present case manipulation was performed at C7 on the right side, as depicted here, and T1 on the left side (not shown).

The chiropractor followed up with the patient three weeks after the final visit, who reported her symptoms were completely absent (i.e., 0 out of 10 on the numeric rating scale). The patient provided written consent for publication of this case and associated images.

## Discussion

We describe a case of chronic NP related to degenerative changes at C5/6 and C6/7 which responded positively and quickly to SMT focusing on the cervicothoracic region coupled with myofascial release. In the current case, periscapular pruritus corresponded to the expected C6 and C7 cutaneous distributions that would correlate with C5/6 and C6/7 degenerative changes identified via radiography. We suggest that the patient’s VKH was unrelated to her NP, which was present prior to the development of VKH and remained unaffected by VKH therapies.

Previous studies have shown that the lower cervical nerve roots provide sensory innervation to the periscapular region [11,12]. Specifically, C6 relates to the angle of the scapula while C7 relates to the interscapular region. In accordance with these studies, we suspect that in the current case, impingement and/or inflammation of the C6 nerve root due to C5/6 disc degeneration and foraminal stenosis led to pruritus in the region of the angle of the scapula. However, this reasoning is based on clinical features alone rather than being supported by advanced imaging or electrodiagnostic testing. Similarly, involvement of the C7 root due to disc degeneration and foraminal stenosis at C6/7 could have led to pruritus in the interscapular region [11,12].

Our findings align with prior research showing that degenerative changes in the cervicothoracic region are associated with NP [1,3]. A previous cross-sectional study identified a significant association between both cervical disc herniation at C6/7, cervical degenerative changes and stenosis, and NP when comparing radiographs and magnetic resonance imaging of those with NP to those with non-NP-related back pain [3]. In the present case, degenerative disc changes may have corresponded with disc bulge or herniation, yet this remains unconfirmed due to our lack of magnetic resonance imaging.

Pruritus relief in the current case may relate to neurophysiologic, biomechanical, or contextual effects of SMT. Chiefly, SMT may provide mechanoreceptive input that inhibits the type of nociceptive transmission involved in pruritus [8]. Second, SMT may have led to the relaxation of paraspinal muscles, thereby diminishing potential entrapment of cutaneous nerve branches. Research showing improvement with SMT of another neuropathic itch disorder, brachioradial pruritus, lends credibility to the idea that SMT could benefit these conditions when related to a spinal etiology [13]. In the present case, the influence of a positive patient expectation for care cannot be excluded, however, lasting improvement despite a treatment break suggests the care had a clinically relevant effect.

We are aware of two prior cases of NP improving with SMT. In one case, a 36-year-old man with a three-year history of bilateral NP and brachioradial pruritus and mild degenerative cervical changes improved following a short course of SMT directed to the cervical and thoracic regions, heat therapy, and stretches [8]. In another case, a 59-year-old woman with a two-year history of NP which began following a motor vehicle collision improved with a single session of SMT focusing on the thoracic and rib articulations [9]. Other nonpharmacologic interventions that have shown promise for NP in case reports include acupuncture and cervical traction [14].

Several limitations of this case report should be noted. Chiefly, as a single case, we cannot conclude that SMT was causative in alleviating symptoms or that the identified treatment response could generalize to other patients with NP. Larger studies are needed to examine the treatment response of SMT compared to usual care (e.g., topical agents, and medications). While the patient underwent extensive tests to evaluate for systemic illness, we lacked magnetic resonance imaging which could have provided greater clarity regarding the degenerative cervical changes. This imaging could have allowed us to evaluate for cervical disc displacement and better characterize the foraminal stenosis. In addition, we lacked electrodiagnostic testing which could have determined whether there was firm evidence of radiculopathy. It is also possible that electrodiagnostic testing would have been normal given the absence of neurologic deficits on examination and rapid response to SMT. Effectively, our consideration that the C6 and C7 nerve roots contributed to the patient’s symptoms was solely based on clinical features and the presence of degenerative changes on cervical radiographs alone. We did not obtain any standardized measure of itch such as the Pruritus Severity Scale. Effects of care could have partially been attributed to myofascial release in addition to SMT. However, myofascial release was predominantly applied to muscles outside of the zone of pruritus, whereas SMT targeted the joints immediately sub-adjacent to the affected C6/7 level. The duration of follow-up was relatively short with only about a month of total observation.

## Conclusions

This case report describes a 54-year-old woman with chronic NP and cervical spondylosis who experienced rapid relief after SMT and myofascial release targeting the cervicothoracic spine. Her distribution of pruritus aligned with C6 and C7 periscapular innervation patterns which correlated with the cervical segments affected by degenerative changes. Our findings are comparable to two prior cases, highlighting that SMT could benefit some NP patients. While this case suggests that SMT may alleviate NP, further research is necessary to examine the effectiveness of SMT for this condition.

## References

[1] Robinson C, Downs E, De la Caridad Gomez Y (2023). Notalgia paresthetica review: update on presentation, pathophysiology, and treatment. Clin Pract.

[2] Kwatra SG, Elmariah S, Chisolm S (2024). United States expert panel consensus on uniform nomenclature and diagnosis for neuropathic pruritus. Itch.

[3] Mülkoğlu C, Nacır B (2020). Notalgia paresthetica: clinical features, radiological evaluation, and a novel therapeutic option. BMC Neurol.

[4] Kim BS, Bissonnette R, Nograles K (2023). Phase 2 trial of difelikefalin in notalgia paresthetica. N Engl J Med.

[5] Masaracchio M, Kirker K, States R, Hanney WJ, Liu X, Kolber M (2019). Thoracic spine manipulation for the management of mechanical neck pain: a systematic review and meta-analysis. PLoS One.

[6] Chu EC, Trager RJ, Lee LY, Niazi IK (2023). A retrospective analysis of the incidence of severe adverse events among recipients of chiropractic spinal manipulative therapy. Sci Rep.

[7] Trager RJ, Dusek JA (2021). Chiropractic case reports: a review and bibliometric analysis. Chiropr Man Therap.

[8] Faye LJ, Budgell BS (2020). Presumptive spondylogenic pruritus: a case study. J Can Chiropr Assoc.

[9] Richardson BS, Way BV, Speece AJ (2009). Osteopathic manipulative treatment in the management of notalgia paresthetica. J Am Osteopath Assoc.

[10] Urzua CA, Herbort CP Jr, Takeuchi M (2022). Vogt-Koyanagi-Harada disease: the step-by-step approach to a better understanding of clinicopathology, immunopathology, diagnosis, and management: a brief review. J Ophthalmic Inflamm Infect.

[11] Mizutamari M, Sei A, Tokiyoshi A, Fujimoto T, Taniwaki T, Togami W, Mizuta H (2010). Corresponding scapular pain with the nerve root involved in cervical radiculopathy. J Orthop Surg (Hong Kong).

[12] Tanaka Y, Kokubun S, Sato T, Ozawa H (2006). Cervical roots as origin of pain in the neck or scapular regions. Spine (Phila Pa 1976).

[13] Kavanagh KJ, Mattei PL, Lawrence R, Burnette C (2023). Brachioradial pruritus: an etiologic review and treatment summary. Cutis.

[14] Ansari A, Weinstein D, Sami N (2020). Notalgia paresthetica: treatment review and algorithmic approach. J Dermatolog Treat.

